# Comparison of cutaneous nerve injury and vessel disruption complications following saphenous vein stripping using big or small olive heads

**DOI:** 10.12669/pjms.323.10017

**Published:** 2016

**Authors:** Mustafa Cuneyt Cicek, Omer Faruk Cicek, Gokhan Lafci, Alper Uzun

**Affiliations:** 1 Mustafa Cuneyt Cicek, Department of Cardiovascular Surgery, Konya Numune State Hospital, Konya, Turkey; 2 Omer Faruk Cicek, Department of Cardiovascular Surgery, Turkiye Yuksek Ihtisas Education and Research Hospital, Ankara, Turkey; 3 Gokhan Lafci, Department of Cardiovascular Surgery, Turkiye Yuksek Ihtisas Education and Research Hospital, Ankara, Turkey; 4 Alper Uzun, Department of Cardiovascular Surgery, Turkiye Yuksek Ihtisas Education and Research Hospital, Ankara, Turkey

**Keywords:** Varicose veins, surgery, peripheral nerve injury

## Abstract

**Objective:**

To compare the nerve injury and vessel disruption complicaitons in patients undergoing saphenous vein stripping using olive heads of different sizes.

**Methods:**

Big olive heads were used in group A (n=50) and small olive heads were used in group B (n=50) from the ankle to the groin; in group C (n=50), the vein was stripped in two sections; in an upward fashion by stripping the distal portion from the ankle to the level of the knee using small olive heads and by stripping the proximal portion from the knee to the level of the groin using big olive heads.

**Results:**

Six months after the operation, nerve injury symptoms were identified in 26%, 4%, 6% of patients in groups A, B, and C respectively. Vessel disruption occurred 2% in group A, 32% in group B, and 4% in group C. Both vessel disruption and nerve injury complications of group C were significantly lower than group A and B (p<0.001).

**Conclusion:**

Saphenous stripping using big olive heads for the proximal portion from the groin down to the level of the knee and using small olive heads for the distal portion from the knee to the level of the ankle is the alternative method which results in minimal nerve injury and vessel disruption.

## INTRODUCTION

Varicose vein surgery operations include complications such as hematoma, femoral artery injury, deep venous thrombosis, pulmonary embolism and saphenous nerve injury.^1^,^2^ Among these, saphenous nerve injury has long been recognized as a potential complication of greater saphenous vein stripping.^2^ Anatomic studies have identified several reasons why these injuries may occur.^3^,^4^ The saphenous nerve is located adjacent to the greater saphenous vein throughout much of its course. This association is particularly close from several centimeters below the knee to the medial malleolus. The nerve has several branches which are particularly vulnerable to avulsion during upward stripping, as the head used for the stripping procedure can become engaged with the branches and disrupt them.^4^

Retrospective estimates of the incidence of nerve injury when the great saphenous vein is stripped from the groin to the ankle range from 23% to 40%.^2^,^5^ No study has directly addressed the effects of saphenous nerve injury on patients’ quality of life. To avoid this complication many alternative techniques are performed such as high ligation and stab avulsion, radiofrequency ablation, and endovenous laser ablation.^6^-^8^ We therefore performed a study on our patients who had venous stripping surgery to compare the ranges of nerve injury and vessel disruption.

## METHODS

### Study design: 

This study was conducted in accordance with the policies and procedures of the Training and Planning Committee of our hospital. Written informed consent was obtained from all the patients.

One hundred fifty patients who underwent varicose vein surgery by the same surgeon at our clinic were included in the study. Patients’ data were collected from the hospital records retrospectively. Patients’ preoperative clinical statuses were classified according to Clinical Severity, Etiology or Cause, Anatomy, Pathophysiology (CEAP) classification (Table-I). The presence of sapheno-femoral reflux and diameters of the great saphenous vein above the level of the knee were confirmed by two-dimensional ultrasound examination (model SSA-770A ultrasound system; Toshiba, Tokyo, Japan). Diameter of the great saphenous veins at the thigh and below the knee levels were greater than 6 and 4 mm and were especially dilated much more around the origins of the perforator veins. The varicose veins were marked preoperatively for all patients. Patients excluded from the study were; those unable or unwilling to participate, patients with a history of surgery to the limb to be operated on and patients with abnormal neurological findings at preoperative assessment (e.g. due to previous trauma, ulceration or diabetic neuropathy).

**Table-I:** Demographic and baseline characteristics of the patients.

	*Group A (N=50)*	*Group B (N=50)*	*Group C (N=50)*	*p*
Age (years; mean±SD)	36.16+12.31	36.14+11.00	40.18+17.01	>0.05
*Gender n (%)*				
Male	26 (52%)	25 (50%)	23 (55.5%)	>0.05
Female	24 (48%)	25 (50%)	27 (44.5%)
*Preoperative CEAP Class*				
Class 0	0	0	0	n/a
Class 1	0	0	0	n/a
Class 2	25	30	29	>0.05
Class 3	16	15	12	>0.05
Class 4	9	5	9	>0.05
Class 5	0	0	0	n/a
Class 6	0	0	0	n/a

The patients were divided into three groups according to the size of olive heads used for stripping and the sections where greater saphenous veins drawn out. In group A (n=50) big olive heads were used for stripping and the vein was drawn out from the ankle incision. In group B (n=50) small olive heads were used for stripping and the vein was drawn out from the ankle incision also. In group C (n=50), the vein was stripped in two sections in an upward fashion, by stripping the distal portion from the ankle to the level of the knee using small olive heads. In all patients, the greater saphenous veins were stripped using conventional technique. At the 6 month follow up, each patient was interviewed and examined by the surgeon. Patients were asked specifically about pain, numbness, tingling, burning, altered sensation and weakness.

### Demographics

One hundred and fifty patients were enrolled in this study. The median age of the patients was 49 years (range; from 25 to 67). Sixty four (42.7%) patients were female. Demographic characteristics of patients are presented in Table-I. Ninety four patients (56%) were classified as Grade-2, forty patients (26.7%) were classified as Grade-3 and twenty six patients (17.3%) were classified as Grade-4 according to CEAP Classification.

### Surgical procedure

All of the procedures were performed in the supine position by the same surgeons under spinal or general anesthesia. Surgery was undertaken through a small skin incision transversally above the inguinal crease and over the sapheno-femoral junction. Tributaries were ligated with 00 Vicryl (Ethicon, UK) and then the great saphenous vein was divided and ligated at its junction. A vein stripper (VastripTM, Astratech, Sweden) was introduced into the cut vein from the ankle upwards and passed through the entire length of the vein. Small olive heads were replaced by big olive heads above the level of the knee through a small incision and the proximal portion was stripped from the knee to the level of the groin using big olive heads in Group C. For all the patients, lengths of the stripped great saphenous veins were checked intraoperatively to see whether the entire length of vein had been extracted or not. If the entire length of the great saphenous vein was not extracted, this was defined as vessel disruption. Secondary procedures were not applied due to shortness of the remaining segments. The subcutaneous tissue of the groin incision was closed using 00 Vicryl (Ethicon, UK) and the skin was closed using 000 Monocryl (Ethicon, UK).

### Statistical analysis

Data analyses were performed by using SPSS for Windows, version 11.5 (SPSS Inc., Chicago, IL, USA). Continuous variables were shown as mean±standard deviation, along with their ranges. Statistical analysis was performed by constructing contingency tables, using Kolmogorov-Smirnov testing, p< 0.05 was considered to be statistically significant.

## RESULTS

There were no significant difference between the groups with respect to demographic data and preoperative CEAP classification. In group A, only one vessel disruption (2%) occurred during the surgery and at the examination 6 months after the operation, nerve injury symptoms were identified in 13 limbs (26%). In group B, vessel disruption during the surgery was seen in 16 limbs (32%) and on examination 6 months postoperatively; nerve injury symptoms were identified in two limbs (4%). Finally in group C, vessel disruption during the surgery was seen in two limbs (4%) and on examination 6 months postoperatively, nerve injury symptoms were identified in three limbs (6%) (Table-II). Both vessel disruption and nerve injury complications of group C were significantly lower than group A and B (p<0.001).

**Table-II:** Results of surgery for varicose veins.

	*Recurrent Varices*	*Nerve Injury*	*Vessel Disruption*
Group A (Big olive head)	-	13 (26%)	1 (2%)
Group B (Small olive head)	-	2 (4%)	16 (32%)
Group C (Big+small olive heads)	-	3 (6%)	2 (4%)
P value	-	p<0.001	p<0.001

## DISCUSSION

Varicose veins and their treatment have been commented upon since antiquity. Although the surgical treatment of ligation and stripping of the greater saphenous veins has been fairly standard for almost the past 100 years,^9^ more recent studies have questioned this approach.^10^ The simplest surgical procedure is ligation, which involves tying off the enlarged vein in portions of the leg, thigh, and groin. Phlebectomy and stripping are probably the best known procedures; however, they are more of a collection of procedures than single techniques.^11^ Recently, traditional surgical techniques have been developed, changed and modified to decrease the potential complications. For example, endoscopic techniques for perforator ligation or endovascular therapies have become very prevalent and also subcutaneous infiltration of tumescent anesthesia with hydrodissection, and a powered phlebectomy catheter to extract multiple branch varices through limited incisions have been developed. Combinations of conservative measures and more invasive techniques may be appropriate, depending on the patient’s symptoms, the extent of vascular pathology, and the available resources. For example, 12-month ulcer recurrence rates are significantly reduced in patients treated with compression and surgery compared with those treated with compression alone.^12^-^14^ A specific combination or standard protocol cannot currently be recommended. All of these approaches have some limitations. Therefore, we modified the traditional great saphenous vein stripping technique and showed the successful results of this simple technique in our study.

In contrast to some descriptions of proximal segment of the procedure, we prefer to make the skin incision transversally above the inguinal crease. A small incision can be made starting over the femoral artery and continuing laterally above the inguinal crease for approximately 5 cm. In obese patients this skin incision would be longer. There are several advantages to placement of the skin incision in this area; i) it is cosmetically pleasing (we know that some young patients want surgery for cosmetic causes), ii) the incision is under minimal tension, iii) it places the incision over the usual location of the sapheno-femoral junction.

The distal saphenous vein is then identified at the knee. It is reported that stripping at the knee rather than at the ankle reduces the risk of saphenous nerve injury, since the saphenous nerve typically joins the saphenous vein in the mid- to upper calf. In our study, in group C, removing the high portion of the vein from the knee incision with big olive heads, and switching to a small olive stripper heads for the below knee portion of the procedure substantially reduces the bulk of the tissue that is pulled through the leg and could potentially reduce the risk of significant trauma to the nerve. We recommend this approach if complete greater saphenous vein stripping is desired.

Flush ligation and stripping of the greater saphenous vein, as advocated by Myers nearly 50 years ago,^15^ has been accepted as the standard management for symptomatic varicose veins. However, since this procedure removes one of the most valuable conduits for arterial bypass operations, and results in significant saphenous vein injury, thigh hematomas, and discomfort, many have argued for preservation of the great saphenous vein.^10^

Selective preservation of the great saphenous vein has been shown to reduce significantly the incidence of nerve injury from 23% to 40% to less than 5%.^16^ However, abundant data from prospective randomized trials indicate that a uniform policy of the great saphenous vein preservation is associated with an unacceptably high rate of recurrent reflux and varicosities. Sorrentino et al.^17^ used an invagination technique for stripping of the great saphenous vein and the range of nerve injury was 1.5% without any vessel disruption in their study.

According to our results, we recommend that great saphenous vein stripping using big olive heads for the proximal portion, from the groin to the level of the knee, and using small olive heads for the distal portion from the knee to the level of the ankle. This is the alternative method to prevent saphenous nerve injury and vessel disruption complications, especially in developing countries where conventional techniques are still being used in practice.
